# The neuroprotective effects of *Tao*-*Ren*-*Cheng*-*Qi Tang* against embolic stroke in rats

**DOI:** 10.1186/s13020-017-0128-y

**Published:** 2017-01-31

**Authors:** Ling-Wei Hsu, Wei-Cheng Shiao, Nen-Chung Chang, Meng-Che Yu, Ting-Lin Yen, Philip Aloysius Thomas, Thanasekaran Jayakumar, Joen-Rong Sheu

**Affiliations:** 1 Graduate Institute of Medical Sciences, College of Medicine, Taipei Medical University, Taipei, Taiwan; 2 Department of Internal Medicine, Yuan’s General Hospital, Kaohsiung, Taiwan; 3 Department of Internal Medicine, School of Medicine, Taipei Medical University, Taipei, Taiwan; 4 Department of Microbiology, Institute of Ophthalmology, Joseph Eye Hospital, Tiruchirappalli, Tamil Nadu 620 001 India; 5 Department of Pharmacology, School of Medicine, Taipei Medical University, Taipei, Taiwan

## Abstract

**Background:**

Combinations of the traditional Chinese and Western medicines have been used to treat numerous diseases throughout the world, and there is a growing body of evidence showing that some of the herbs used in traditional Chinese medicine elicit significant pharmacological effects. The aim of this study was to demonstrate the neuroprotective effects of *Tao*-*Ren*-*Cheng*-*Qi Tang* (TRCQT) in combination with aspirin following middle cerebral artery occlusion (MCAO)—induced embolic stroke in rats.

**Methods:**

A blood clot was embolized into the middle cerebral artery of rats to induce focal ischemic brain injury. After 24 h of MCAO occlusion, the rats were arbitrarily separated into five groups and subjected to different oral treatment processes with TRCQT and aspirin for 30 days before being evaluated in terms of their neurological behavior using a four-point system. The rats were sacrificed at 30 days after drug treatment and the infarct volumes were measured using a 2,3,5-triphenyltetrazolium chloride staining method. Tumor necrosis factor-α (TNF-α), c-Jun N-terminal kinases (JNK), activated caspase-3 and Bax were detected by western blot analysis. The apoptotic cells were identified by Terminal deoxynucleotidyl transferase dUTP nick end labeling (TUNEL) staining. ROS generation was also measured by electron spin resonance spectrometry.

**Results:**

Rats treated with TRCQT alone or in combination with aspirin showed a significantly reduced infarct volume (*P* < 0.001) and improved neurological outcome compared with those treated with distilled water. Rats treated with TRCQT alone (*P* = 0.021) or in combination with aspirin (*P* = 0.02) also showed significantly reduced MCAO-induced expression levels of TNF-α and pJNK (*P* < 0.001) in their ischemic regions. Rats treated with TRCQT alone or in combination with aspirin showed decreased apoptosis by a reduction in the number of TUNEL positive cells, which inhibited the expression of activated caspase-3 (*P* = 0.038) and Bax (*P* = 0.004; *P* = 0.003). TRCQT also led to a significant concentration-dependent reduction in the formation of hydroxyl radicals (*P* < 0.001).

**Conclusions:**

TRCQT reduced brain infarct volume and improved neurological outcomes by reducing apoptosis, attenuating the expression of TNF-α and p-JNK, and reducing the formation of hydroxyl radicals in MCAO-induced embolic stroke of rats.

**Electronic supplementary material:**

The online version of this article (doi:10.1186/s13020-017-0128-y) contains supplementary material, which is available to authorized users.

## Background

The incidence of stroke has markedly increased in developing countries over the past four decades [1]. Most strokes are caused by a thromboembolism, which disrupts cerebral blood flow, leading to oxygen and glucose deprivation in cells [2]. Secondary strokes who the individuals have a stroke history often have a higher rate of death and disability because of existing damage to the parts of the brain injured by the original stroke. Aspirin has been used to prevent secondary stroke. The incidence of cerebral hemorrhage and other bleeding events is higher in China than it is in people from other high-income countries [3]. Ischemic brain tissue can be partially prevented from sprouting into infarction by several neuroprotective drugs [4]. It is noteworthy however, that numerous experimentally effective neuroprotective drugs have failed in clinical trials in human because of serious side effects [5].

Chinese medicines (CMs) usually contain a large number of compounds, which can affect multiple targets [6, 7], and several CMs have been used to treat stroke [8]. Previous studies have reported that the well-known Chinese formula *Tao*-*Hong*-*Si*-*Wu Tang* can be used to treat type 2 diabetes [9], as well as several mental disorders, including periodic psychosis, mania, neurosis, menopausal syndrome and involutional depression [10]. However, *Tao*-*Ren*-*Cheng*-*Qi Tang* (TRCQT) has not been clinically or experimentally used to treat stroke. In this study, we have used TRCQT, which consists of *Tao Ren* (*Prunus persica* (L.) Batsch., 5.0 g), *Gui Zhi* (*Cinnamon Twig.,* 5.0 g), *Mang Xiao* (*Natrii Sulfas,* 5.0 g), *Zhi Gan Cao* (*Radix Glycyrrhizae Preparata.,* 5.0 g) and *Da Huang* (*Radix et Rhizoma Rhei*.,10 g), to treat ischemic stroke in a rat animal model. The aim of this study was to demonstrate the neuroprotective effects of TRCQT in combination with aspirin following a middle cerebral artery occlusion (MCAO)-induced embolic stroke in rats.

## Methods

### Tao-Ren-Cheng-Qi Tang

A dried powder sample of TRCQT (Batch Number, 161342) was purchased from the Sun Ten Pharma. Co. (Taichung, Taiwan). The composition of a 30-g portion of this material was as follows: *Tao Ren* 5.0 g, *Gui Zhi* 5.0 g, *Mang Xiao* 5.0 g, *Da Huang* 10 g, *Zhi Gan Cao* 5.0 g. Thirty grams of this herbal mixture was extracted with water, yielding 7.0 g of dry extract (30.0:7.0 = 4.3:1). This material was mixed with 5 g of corn starch to give 12 g of final product. For sample preparation, the dried extract of TRCQT was dissolved in sterilized saline water (0.9% NaCl) at a concentration of 0.5 g/kg.

### Reagents

3-(4,5-Dimethylthiazol-2-yl)-2,5-diphenyltetrazolium bromide (MTT) and propidium iodide (PI) were purchased from Sigma-Aldrich (St. Louis, MO, USA). TNF-α, JNK, Bax and activated caspase-3 antibodies were purchased from Cell Signaling Technology (Beverly, MA, USA). The anti-α-tubulin mAb was purchased from Neo Markers (Fremont, CA, USA). The Hybond-P polyvinylidene difluoride membrane, enhanced chemiluminescence (ECL) western blotting detection reagent and analysis system, horseradish peroxidase (HRP)-conjugated donkey anti-rabbit immunoglobulin G (IgG) and sheep anti-mouse IgG were purchased from Amersham (Buckinghamshire, UK).

### MCAO-induced ischemia rat model

Healthy male Wistar rats (250–300 g) were used in this study. All of the animal studies were conducted in accordance with the standards established in the Guide for the Care and Use of Laboratory Animals, which was published by the Institutional Animal Care and Use Committee (IACUC) of Taipei Medical University (Additional file 1). The animal studies were also performed in accordance with the ARRIVE guideline (Additional file 2). Thirty rats were acclimated for 20 days before dosing, but only 25 of these animals were used the following experiments. All of the rats were kept at 37 °C in groups of five under a 12-h dark/light cycle with ad libitum access to food and water before the surgery. The rats were subjected to MCAO-induced ischemia by the administration of an autologous blood clot, as described in our previous studies [11, 12]. After surgery, the rats were housed individually under similar environmental conditions and were found to be free of apparent infection or inflammation, as well as showing no neurological deficits. In this study, at 24 h after MCA occlusion, the rats were arbitrarily separated into five groups of five rats each, including (group 1) sham-operated; (group 2) orally treated with distilled water for 30 days, followed by thromboembolic occlusion; (groups 3 and 4) treated with aspirin (5 mg/kg) and TRCQT (0.5 g/kg) alone for 30 days, followed by thromboembolic occlusion; and (group 5) treated with TRCQT (0.5 g/kg) combined with aspirin (5 mg/kg), followed by thromboembolic occlusion.

### Neurological functional tests

To assess the neurobehavioral scoring of the animals, a sensorimotor integrity was measured for 30 days after MCAO by an investigator with no prior knowledge of the group allocation (Additional file 3) [13]. The neurologic scores were measured by using a 4-point sliding scale. Each rat was examined for resistance to lateral push (score = 4), open field circling (score = 3) and shoulder adduction (score = 2) or contralateral forelimb flexion (score = 1) when held by the tail. Rats extending both forelimbs towards the floor and not showing any other signs of neurologic impairment were scored 0. In this study, all of the rats subjected to MCAO either exhibited a neurologic score of 4 when they were examined 24 h after ischemia or immediately before reperfusion, or were excluded from the study.

### Quantification of brain infarct volume

At 24 h after reperfusion, the rats were anesthetized and their brains were removed and cut into 2-mm-thick slices. The slices were then immersed in a 2% solution of 2,3,5-triphenyltetrazolium chloride (TTC) in phosphate-buffered saline at 37 °C for 30 min and fixed in 4% phosphate buffered formalin. The infarct areas of each slice were determined using a computerized image analyzer (Image-Pro plus). These areas were then summed and multiplied by the slice thickness to give the infarct volumes of each slice, which were calculated according to the method reported by Hsiao’s group [11]. None of the animals died during these experiments.

### Western blot analysis

After 30 days of TRCQT treatment followed by thromboembolic occlusion, the rat brain tissues were collected and homogenized, followed by sonication in a lysis buffer containing 20 mM Tris–HCl (pH 7.5), 1 mM MgCl_2_, 125 mM NaCl, 1% Triton X-100, 1 mM phenylmethylsulfonyl fluoride, 10 μg/mL leupeptin, 10 μg/mL aprotinin, 25 mM β-glycerophosphate, 50 mM sodium fluoride and 100 μM sodium orthovanadate. The expression levels of TNF-α, phospho-JNK, activated caspase-3 and Bax-2 were analyzed by immunoblotting, as described previously by Rodrigo et al. [14] with minor modifications. A mixture of tris-buffered saline and Tween 20 (TBST) buffer containing 0.1% Tween 20 was used to wash the membranes.

### Detection of DNA fragmentation by TUNEL assay

The in situ detection of DNA fragmentation in the brain tissues of the rats was performed 30 days after TRCQT treatment followed by thromboembolic occlusion using a TUNEL detection kit (Millipore, Billerica, Massachusetts, USA). Briefly, brain tissues were fixed in 4% formaldehyde and embedded in paraffin wax. Five micrometer sections of the embedded material were then cut and washed for 30 min in PBS. The sections were then equilibrated in water for 30 min at 37 °C, before being incubated in a mixture of Proteinase K (250 µL) enzyme at 37 °C for 30 min. The proteinase K digestion process was stopped by washing the sections with PBS (four 2-min wash cycles). The sections were then incubated in terminal deoxynucleotidyl transferase (TdT) buffer [2.5 mM Tris–HCl (pH 6.6), 0.2 M potassium cacodylate, 2.5 mM CoCl_2_, 0.25 mg/mL bovine serum albumin (BSA)] for 10 min, followed by 60 min at 37 °C in a TdT end-labeling cocktail. The TdT end-labeling cocktail was removed and the reaction was stopped, and the resulting sections were washed in PBS. The sections were incubated in Avidin-FITC in the dark for 30 min at 37 °C and then washed in PBS to end the reaction. The sections were then counterstained with PI for 10 min at 37 °C and mounted on a cover slip. Only the green-stained cells were counted as belonging to the apoptotic phenotype and the density of apoptotic cells was detected by immuno-fluorescent microscopy (TCS SP5, Leica, Mannheim, Germany) by counting the green-stained cells.

### Measurements of hydroxyl radical (OH^·^^−^) formation by electron spin resonance (ESR) spectrometry

ESR experiments were conducted according to the method reported by Chou et al. [15] using a Bruker EMX ESR spectrometer (Billerica, MA, USA). Briefly, a Fenton reaction solution (50 µm FeSO_4_ + 2 mM H_2_O_2_) was pretreated with a solvent control (0.1% DMSO) or TRCQT (0.3, 0.7 and 1.3 mg/mL) for 10 min. The rate of hydroxyl radical-scavenging activity was defined by the following equation:

$$\begin{aligned} &{\text{Inhibition rate }} = \\ &\quad { 1} - \left[ {{\text{signal height }}\left( {\text{TRCQT}} \right)/{\text{signal height }}\left( {\text{control}} \right)} \right] \end{aligned}$$

### Statistical analysis

The results were expressed as the mean ± SD and were accompanied by the number of observations. The experiments were assessed by analysis of variance (ANOVA), version 9.2 (SAS Inc., Cary, NC, USA), using the Newman–Keuls method. A *P* value of less than 0.05 was considered statistically significant. Concentration-dependent effects were determined by visual inspection of the results.

## Results

### Effect of TRCQT, aspirin alone, or in combination on infarct volume

**Fig. 1:**
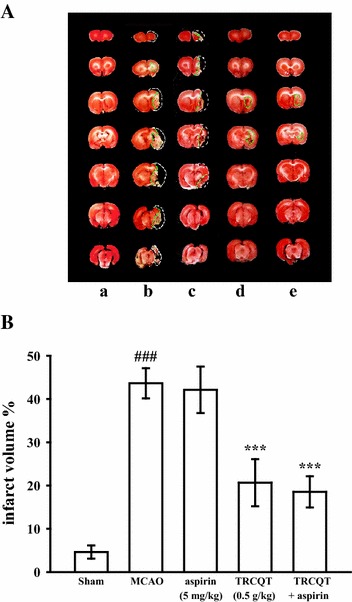
Effects of TRCQT with aspirin against thromboembolic stroke in rats. The infarct volume was measured after 30 days of treatment with TRCQT, aspirin or a combination of TRCQT and aspirin after middle cerebral artery occlusion (MCAO). **A** TTC (2,3,5-triphenyltetrazolum chloride solution) staining of the brain slices showing the infarct areas in the sham (*a*), thromboembolic occlusion-induced untreated (*b*), aspirin (5 mg/kg) (*c*), TRCQT (0.5 g/kg) (*d*) and combination-treated groups (*e*). **B** Histogram showing the quantification of the infarct volumes in the sham, thromboembolic occlusion-induced untreated, aspirin, TRCQT and combination-treated groups. These data represent the mean ± SD of three independent experiments. ^###^ *P* < 0.001 compared with sham group, ***P < 0.001 compared with the thromboembolic occlusion-induced untreated group (*n* = 5)

The effects of TRCQT and aspirin alone or in combination on the infarct volumes are shown in Fig. 1A, B. Compared with the group treated with distilled water (group 2), the animals treated with TRCQT alone showed a significant decrease in their infarct volume (*P* < 0.001). In contrast, the group treated with aspirin alone (group 3) did not show any significant effects. A similar effect was also found in rats treated with aspirin combined with TRCQT (group 5) (*P* < 0.001) for 30 days after MCAO compared with the sham-operated (group 1).

### Neurobehavioral assessment

**Fig. 2:**
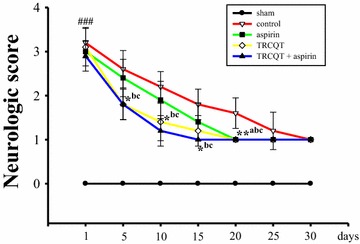
Effects of TRCQT and aspirin on the neurological deficit. The panel shows the neurological deficit scores after stroke in the five experimental groups: sham, thromboembolic occlusion-induced untreated, aspirin, TRCQT and combination-treated. These data represent the mean ± SD of three independent experiments. ^###^ *P* < 0.001 compared with the sham group, *P < 0.05 compared with the thromboembolic occlusion-induced untreated group (*n* = 5)

Neurological deficit was examined at 24 h after reperfusion and scored on a 4-point scale according to the method described by Bederson’s group [13]. The changes observed in the neurological deficit scores of the different groups are shown in Fig. 2. Rats treated with aspirin (group 3) for 30 days after MCAO exhibited a moderate reduction in their neurobehavioral scores compared with the sham-operated group at 24 h after ischemia (*P* = 0.0525). However, rats treated with TRCQT alone (group 4) for 30 days showed a significant reduction in their neurobehavioral deficit score compared with the sham-operated group at 24 h after thromboembolic occlusion (*P* = 0.0537). Notably, rats treated with a combination of aspirin and TRCQT showed a better neurobehavioral outcome than those treated those treated with TRCQT or aspirin alone (*P* = 0.0119).

### TRCQT enhanced aspirin’s inhibitory effect on TNF-α and p-JNK in the ischemic brain

**Fig. 3:**
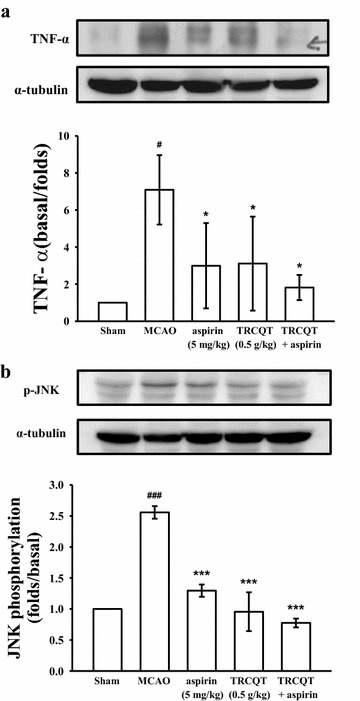
Effects of TRCQT combined with aspirin on the expressions of TNF-α (**a**) and p-JNK (**b**) in cerebral homogenates 30 days after thromboembolic stroke in rats. The data represent the mean ± SD of three independent experiments. ^#^ and ^###^ *P* < 0.01 compared with the sham-operated group, * and ***P < 0.05 compared with the thromboembolic occlusion-induced untreated group. Equal loading in each lane was demonstrated by similar intensities of α-tubulin (*n* = 5)

As shown in Fig. 3a, there was a significant increase in the expression levels of TNF-α in the MCAO-induced group, whereas the groups treated with aspirin (*P* = 0.043) or TRCQT (*P* = 0.021) alone showed significant decreases in the expression of this protein. Moreover, a significant inhibitory effect was observed in the expression of TNF-α in the rats treated with aspirin (0.5 g/kg) combined with TRCQT (*P* = 0.02).

The effects of the TRCQT, aspirin and TRCQT/aspirin combination on the levels of p-JNK are shown in Fig. 3b. Compared with the MCAO-induced group, the rats treated with TRCQT or aspirin alone showed a significant decrease in their p-JNK levels (*P* < 0.001). The rats treated with a combination of aspirin and TRCQT also showed a significant decrease in their expression of p-JNK compared with those from the individual treatment groups (*P* < 0.001).

### TRCQT inhibited the apoptosis-related proteins Bax and activated caspase-3 in the ischemic brain tissues

**Fig. 4:**
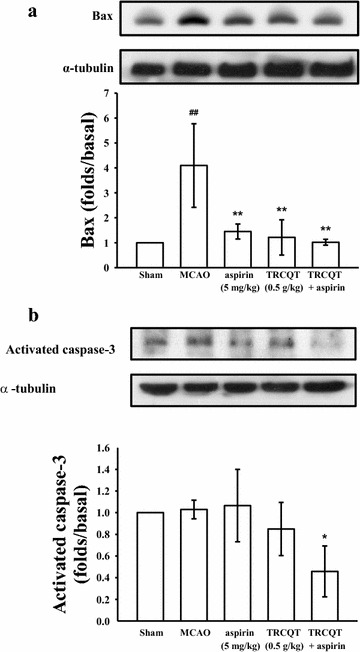
Effects of TRCQT combined with aspirin on the expression levels of Bax (**a**) and activated caspase-3 (**b**) in cerebral homogenates 30 days after thromboembolic stroke in rats. The data represent the mean ± SD of three independent experiments. ^##^Compared with the sham-operated group, **compared with the thromboembolic occlusion-induced untreated group. Equal loading in each lane was demonstrated by similar intensities of α-tubulin (*n* = 5)

As shown in Fig. 4a, there was a significant increase (*P* = 0.015) in the levels of Bax protein in the MCAO-induced group compared with the sham group. Although the rats treated with aspirin or TRCQT alone showed a significant decrease in the expression of Bax (*P* = 0.018 and 0.013, respectively), this effect was potentiated when these treatments were combined (*P* = 0.011).

The expression of activated caspase-3 did not change in the sham, MCAO-induced or aspirin treated rats (Fig. 4b), but there was significant decrease in the expression levels of this protein in the rats treated with a combination of aspirin and TRCQT (*P* = 0.048). The rats treated with TRCQT alone also showed a considerable decrease in their activated caspase-3 expression, although this effect was not significant.

### Effect of combination of TRCQT and aspirin on DNA fragmentation

**Fig. 5:**
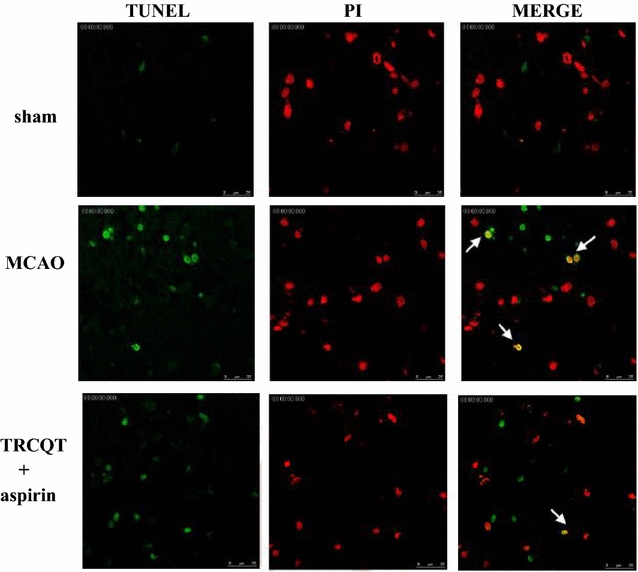
Effects of a combination of TRCQT and aspirin on DNA fragmentation after MCAO injury in rats. TUNEL staining images of damaged cortex samples collected from each group after TRCQT combined with aspirin administration at high magnification (25 µm). The TUNEL-positive material was localized in the nuclei of the neurons. Numerous TUNEL-positive cells were identified in the thromboembolic occlusion-induced untreated group compared with the sham group and the group treated with a combination of TRCQT and aspirin

Although no TUNEL-positive cells were identified in the sham-operated group (Fig. 5), we did observe a considerable increase in the number of TUNEL-positive cells in the MCAO-induced group. Rats treated with a combination of TRCQT and aspirin showed a noticeable reduction in the number of TUNEL-positive cells, although this effect was not statistically significant.

### TRCQT attenuated the in vitro formation of HO· radicals

**Fig. 6:**
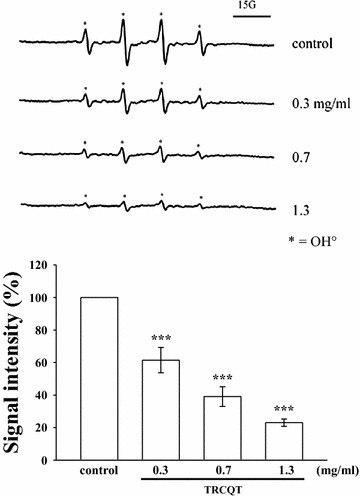
Effects of TRCQT on the formation of hydroxyl radicals; the ESR spectra showed that TRCQT significantly inhibited the formation of hydroxyl radicals at concentrations of at 0.3, 0.7 and 1.3 mg/mL in the Fenton reaction. These data represent the mean ± SD of three independent experiments. ***P < 0.001 compared with the control group (*n* = 3)

*TRCQT* significantly attenuated the Fenton reaction-induced formation of HO· radicals in a concentration-dependent manner (0.3, 0.7 and 1.3 mg/mL) (Fig. 6). Moreover, TRCQT inhibited the formation of HO· radicals to a much greater extent at a concentration of 1.3 mg/mL compared with a concentration of 0.3 mg/mL (*P* < 0.001).

## Discussion

This study demonstrated that the oral treatment with TRCQT or aspirin suppressed thromboembolic stroke in rats and led to an improved neurological outcome by reducing the infarct volume. The treatment of rats with TRCQT or aspirin alone inhibited the expression of TNF-α, p-JNK, activated caspase-3 and Bax, as well as reducing the number of TUNEL positive cells in the ischemic brain regions of these animals. Although the treatment of the rats with TRCQT or aspirin alone resulted in neuroprotective effects, the combination of these two drugs was much more effective. Treatment with TRCQT (0.3, 0.7 and 1.3 mg/mL) led to a significant reduction in the formation of HO· radicals.

Vascular inflammation is mediated by pro-inflammatory cytokines, such as TNF-α [16, 17]. The administration of TNF-α leads to an increase in tissue damage and neurological deficits, thereby aggravating ischemic brain injury [18]. Anti-TNF neutralizing antibodies [19] and the inhibition of soluble TNF-α receptor type 1 [20] have been reported to reduce ischemic damage and improve functional outcome after stroke [21]. The results of our previous study demonstrated that the oral administration of *Tao*-*Hong*-*Si*-*Wu Tang* (THSWT) attenuated the effects of embolic stroke by inhibiting the expression of TNF-α [22]. In this study, the treatment of MCAO-induced rats with TRCQT or aspirin alone led to a reduction in the TNF-α levels. Cerebral ischemia results in an increase in the levels of mitogen activated protein kinases (MAPKs) [23, 24]. Inhibition of the JNK MAPK pathways could therefore improved outcomes in ischemic brain injury by suppressing the production of inflammatory cytokines [25, 26]. The phosphorylation of JNK can promote stress-induced cell death and regulate the downstream signaling events associated with apoptosis during ischemic injury [27]. In this study, we observed increases in the activation of TNF-α and p-JNK in rats with MCAO-induced brain injury. Notably, the treatment of these rats with TRCQT or aspirin alone led a significant decrease in the activation of these proteins.

Activated caspase-3 occurs following apoptosis in the hippocampus after transient cerebral ischemia-mediated neuronal death [28, 29]. The levels of activated caspase-3 and Bax observed at 30 days after ischemia were higher than those found in the sham rats. Furthermore, the number of TUNEL-positive cells found in the ischemic region increased after MCAO ischemia. The oral administration of TRCQT in combination with aspirin led to significant decreases in the expression levels of Bax and activated caspase-3, as well as a reduction in the number of TUNEL-positive cells.

The production of reactive oxygen species (ROS) is a significant factor in the neuropathology of stroke [30]. During acute ischemic stroke, ROS can be generated through multiple injury mechanisms, including mitochondrial inhibition, Ca^2+^ overload and reperfusion injury [31]. H_2_O_2_ is the source of HO· radicals, which can contribute to neuronal injury after cerebral ischemic reperfusion, resulting in neuronal death [32, 33]. The hydroxyl radical-scavenging activity of TRCQT was directly evaluated using an ESR method, and the results revealed that TRCQT scavenged HO· radicals in a concentration-dependent manner.

The screening of traditional Chinese medicines to identify potential neuroprotective agents by evaluating their neuroprotective effects using in vivo and in vitro experimental models of stroke is highly desired. A large amount of clinical information has been gathered over the years pertaining to the use of Chinese medicines treat stroke using indigenous herbal materials [34]. The results of this study should provide researchers with a better understanding of the mechanisms underlying the therapeutic value of using a combination of TRCQT and aspirin to treat thromboembolic stroke.

## Conclusions

TRCQT reduced brain infarct volume and improved neurological outcomes by reducing apoptosis, attenuating the expression of TNF-α and p-JNK, and reducing the formation of HO· radicals in MCAO-induced embolic stroke of rats.

## Additional files

**Additional file 1.** The affidavit of approval of animal use protocal.

**Additional file 2.** The ARRIVE guidelines: Animal research; reporting of in vivo experiments.

**Additional file 3. Table:  ** Neurological examination grading system.

## Abbreviations

CCA: common carotid artery
ESR: electron spin resonance spectrometry
ICA: internal carotid artery
JNK: c-Jun N-terminal kinases
MCAO: middle cerebral artery occlusion
MTT: 2,5-diphenyl tetrazolium bromide
OH^−^: hydroxyl radical
PA: pterygopalatine artery
ROS: reactive oxygen species
TdT: terminal deoxynucleotidyl transferase
TNF-α: tumor necrosis factor-α
TRCQT: *Tao*-*Ren*-*Cheng*-*Qi Tang*
TTC: 2,3,5-triphenyltetrazolium chloride
TUNEL: terminal deoxynucleotidyl transferase dUTP nick end labeling
