# Supplementary material for: The neuroprotective effects of Tao-Ren-Cheng-Qi Tang against embolic stroke in rats
Source: Chin Med. 2017 Jan 31;12:7. doi: 10.1186/s13020-017-0128-y (PMC5286857; doi:10.1186/s13020-017-0128-y)
Supplement: Supplementary file 1 — Additional file 1. The affidavit of approval of animal use protocal. [file 13020_2017_128_MOESM1_ESM.pdf]

# 臺北醫學大學動物實驗管理小組審查同意書
# Affidavit of Approval of Animal Use Protocol
# Taipei Medical University

動物實驗申請表暨同意書編號：LAC-100-0092

計畫申請人：許準榕　　職　稱：教授

單　位：醫學科學研究所　　飼養/應用地點：動物中心/解剖學科灌流室

計畫名稱：桃仁承氣湯及三黃瀉心湯併用 aspirin 治療缺血性腦中風及降低神經行為缺陷之交互作用與引起胃出血、顱內出血、蜘蛛膜下腔出血之風險評估

本計畫之「動物實驗申請表」業經動物實驗管理小組 [v] 實質 [ ] 形式審查通過。
本計畫預定飼養應用之動物如下：

| 動物種類 | 動物數量 | 計畫執行期間 |
|---|---|---|
| rat/wistar | 380 x2 = 760 隻 | 101 年02 月 23 日 至102 年12 月15 日 |
| mice/ICR | 144 隻 | |

The animal use protocol listed below has been reviewed and approved by the Institutional Animal Care and Use Committee（IACUC）.

Protocol Title : Comparative studies of Tao-Ren-Cheng-Qi Tang or San-Huang-Xie-Xin Tang with aspirin on neuroprotection and lowering the neurobehavioral deficits in rat ischemic stroke: evaluation the risks of inducing gastric bleeding, intracerebral hemorrhage, and subarachnoid hemorrhage.

IACUC Approval No：

Period of Protocol：Valid From: 02/23/2012 To: 12/15/2014 （mm/dd/yyyy）

Principle Investigator（PI）: Joen-Rong Sheu

動物實驗管理小組召集人　召集人 楊良友　日期
IACUC Chairman　　Date 3/5/2012

◎◎標示　　欄位由管理小組填寫◎◎
