# Supplementary material for: The neuroprotective effects of Tao-Ren-Cheng-Qi Tang against embolic stroke in rats
Source: Chin Med. 2017 Jan 31;12:7. doi: 10.1186/s13020-017-0128-y (PMC5286857; doi:10.1186/s13020-017-0128-y)
Supplement: Supplementary file 3 — Additional file 3. Table: Neurological examination grading system. [file 13020_2017_128_MOESM3_ESM.docx]

**Neurological Examination Grading System**

Normal grade 0: no observable deficit

Moderate grade1: failure to extend left forepaw fully

Severe grade2: circling to the left

grade3: falling to the left

grade4: not walk spontaneously and

depressed level of consciousness

Lee et al. J Neurosci Res. 2002; 68: 636-45.
